# Impact of Little Cigars and Cigarillos Packaging Features on Product Preference

**DOI:** 10.3390/ijerph182111443

**Published:** 2021-10-30

**Authors:** Ce Shang, James Nonnemaker, Kymberle Sterling, Jessica Sobolewski, Scott R. Weaver

**Affiliations:** 1 Department of Internal Medicine, Medical Oncology, The Ohio State University Wexner Medical Center, Columbus, OH 43017, USA; 2 RTI International, Research Triangle Park, NC 22709, USA; jnonnemaker@rti.org (J.N.); jsobolewski@rti.org (J.S.); 3 Department of Health Promotion and Behavioral Sciences, University of Texas Health Sciences Center, School of Public Health, Dallas, TX 77054, USA; Kymberle.L.Sterling@uth.tmc.edu; 4 Department of Population Health Sciences, School of Public Health, Georgia State University, Atlanta, GA 30302, USA; srweaver@gsu.edu

**Keywords:** cigars, cigarillos, packaging, flavor, quality, price, pack size

## Abstract

Background: We conducted a discrete choice experiment (DCE) among young adult cigarette smokers in the period July–August 2018 to examine their preference for cigarillos in response to various packaging-related attributes, including flavor, flavor description, quality descriptors, pack size, and prices. Methods: A convenience sample of 566 US young adult cigarette smokers aged 18–34, among whom 296 were current little cigar and cigarillo (LCC) smokers, were recruited using Facebook ads and invited to participate in an online (Qualtrics) tobacco survey containing DCE and tobacco use questions. In the experiment, participants chose among two cigarillo products or “neither” (opt-out). Results: We analyzed preferences for LCCs using multinomial, nested, random parameter logit models. Results showed that young adult cigarette smokers preferred grape over menthol, tobacco/regular, and wine flavors; “color only” and “color and text” flavor depictions over text only; “smooth” and “sweet” quality descriptors over “satisfying”; and larger pack sizes and lower prices. Conclusions: Regulating packaging-related features will impact LCC choices among US young adult smokers. FDA regulation over these packaging-related features may impact LCC use among young adult smokers.

## 1. Introduction

Despite the success of tobacco control efforts to reduce cigarette smoking, cigar use in the US has steadily increased. Between 2000 and 2018, US cigar consumption increased by 114%, while cigarette smoking decreased by 46% [1]. Among the three categories into which cigars are classified—premium/large cigars, little filtered cigars, and cigarillos—the latter two are most popular, especially among young adults. According to 2016–2017 data from the Population Assessment of Tobacco Survey (PATH), 36.8% of 18–24-year-olds reported ever cigarillo smoking, 18.9% reported ever little filtered cigar smoking, and 3.3% reported currently smoking cigarillos or little filtered cigars daily or on some days [2]. The prevalence of little cigar and cigarillo (LCC) use is concerning because cigar smoking exposes users to higher levels of nicotine than found in cigarettes and carries significant health risks [3,4]. Moreover, as cigarette taxes and prices continue to rise, smokers may switch to smoke LCCs instead of quitting smoking, particularly young smokers who are more price-sensitive than older adults [5].

The popularity of LCCs among young adults can be attributed to several factors, including the product’s relatively low cost, less stringent regulations, and the wide range of flavors in which they are sold [6,7,8,9]. As marijuana use is a predictor of LCC use among young adults, recent legalizations of recreational marijuana may also increase LCC use among this population [10,11,12]. More than 86% of LCCs on the market are flavored [13], and there is evidence that young adults perceive LCCs in general, and flavored LCCs in particular, as less risky to smoke than other tobacco products [14,15].

Features of product packaging may contribute to young adults’ misconceptions about LCC risk. Research has pointed to industry use of package characteristics, e.g., color, images, and flavor descriptors, to manipulate consumers’ perceptions of the product’s harms and target specific groups [16,17,18,19]. Product design (e.g., filters and smaller pack size) and tobacco quality claims (e.g., “natural”) may also contribute to lower risk perceptions. The text, imagery, and color of flavored LCC packaging may convey the sense that it is a lighter and perhaps healthier product [20,21,22]. Despite prohibition of modified risk descriptors (e.g., “mild”) in cigarette products, several flavored LCC brands contain such descriptors on their packaging, and LCCs have no minimum pack size requirements. For example, the crème-colored packaging of “Black & Mild” (a leading LCC brand) contains “mild” and “natural” text descriptors that may have a significant effect on young adults’ beliefs about the product’s risks [23]. Therefore, LCC packaging is a potentially important regulatory area for the US Food and Drug Administration (FDA) to consider.

In November 2018, the US FDA announced its intention to address the sales of cheap cigars and issued a product standard that would ban any flavored cigar to prevent youth tobacco use [24,25]. Further in April 2021, the FDA announced its plans to issue product standards that will ban menthol as a characterizing flavor in cigarettes and ban all characterizing flavors (including menthol) in cigars. The standard has not been enacted, however. The entire product category of flavored cigars—including those that were “grandfathered” and on the market as of 15 February 2007 and new flavored cigar products on the market after this date [26]—remains available for sale and no further federal action focused on flavored cigars has been taken. Notably, in February 2020, the FDA prioritized enforcement against unauthorized (illegally marketed) flavored, cartridge-based ENDS products (i.e., other than tobacco or menthol flavored) to curb the use of these products among young populations, but no actions were taken against flavored LCCs that are also popular among young people [27].

Within this evolving regulatory context, research is needed to further illuminate the relationships between packaging characteristics and young adult consumers’ perceptions of and preferences for LCC products. Moreover, as tobacco regulation becomes more stringent and other marketing strategies such as TV advertisements are banned both in the US and internationally, tobacco companies and manufacturers may increasingly rely on product packaging as a marketing tool [28,29,30]. It is therefore important to understand how the packaging of LCCs impact products preference and appeals.

Although an increasing number of studies have documented lower risk perceptions of flavored LCC use among young adults [31,32,33], it is unclear how young adult smokers may choose LCCs—a likely substitute for cigarettes [2]. There is one experimental study that examines how cigarillo packaging features (flavor descriptor, color, pack, and warning) influence perceptions of product flavor, taste, smell, and appeal, and that study did not explicitly estimate product preference measured by intention to purchase or rank the importance of the packaging features to guide policy priorities. Moreover, it did not ascertain the impacts of packaging policies that have different strengths (e.g., flavor bans including menthol vs. flavor bans not including menthol) on product appeal or preference [18].

In summary, there is no research to date that has explicitly examined (1) whether flavored LCC packaging-related features (e.g., text, colors, pack size, and price) influence young adult smokers’ LCC preference in terms of choosing or purchasing the product; (2) which flavored LCC packaging-related feature(s) influence(s) product preference; or (3) the impacts of packaging policies that have different strengths—three critically important gaps in the field, which, if filled, will enhance the FDA’s effectiveness to regulate the LCC market.

This study uses a discrete choice experiment (DCE) to examine adult smokers’ preference for cigarillo products in response to various packaging-related attributes. We conducted the experiment among a convenience sample of 566 young adult cigarette smokers in the period July–August 2018. The aim was to understand young adults’ preferences for LCC products based on various packaging characteristics, collecting data that could inform FDA regulatory policy decision-making regarding product standards for LCCs.

## 2. Materials and Methods

### 2.1. Discrete Choice Experiment (DCE)

We conducted a DCE in the period July–August 2018 to examine the effects of flavored cigarillo package attributes on young adult current cigarette smokers’ product preference. We selected young adult current cigarette smokers as this group has elevated risks of LCC smoking [34]. DCE is a stated preference technique increasingly used in the health economics and public health fields to understand product attribute impact on product choices, particularly tobacco [35,36,37,38]. DCEs have the advantage of generating hypothetical data when observational data are lacking and inferring causal impact by controlling for unobservable heterogeneity and confounding factors [39]. Recent studies on alcohol and tobacco have further demonstrated the capacity of DCE to predict policy impact before policies are implemented [31,40].

Unlike lab-based experiments, DCEs use survey questions that elicit consumer preference and thus are usually conducted as part of an online survey [35,36,37,38,39,40,41,42]. In this study, young adult cigarette smokers were invited to participate in the online DCE experiment, where they were shown images of hypothetical cigarillo packages that were varied with respect to flavor, flavor depiction (pack color/text), text descriptor of the LCC’s quality, pack size, and price. After viewing the hypothetical packages, the respondents were asked to select their preferred product.

#### 2.1.1. Eligibility Criteria and Recruitment

In the period July–August 2018, we recruited a convenience sample of 566 US young adult cigarette smokers aged 18–34 using Facebook ads, among whom 296 (52.3%) were current LCC smokers (i.e., those who answer that they are smoking LCCs now). Because young adult current cigarette smokers have elevated risks of LCC smoking and may switch from cigarettes to LCCs as cigarette regulation becomes more restricted (e.g., increasing cigarette taxes), we aimed to obtain a sample of cigarettes smokers where half were current LCC users and half were not [34]. Ads were targeted to users based on age (18–34), location (United States), and language (English). To recruit cigarette smokers who currently smoked LCCs, various interest-based keywords were also used (e.g., Black & Mild, cigar, and cigarette). Facebook ads were displayed to users on desktop and laptop computers (sidebar or News Feed), as well as smartphones (mobile News Feed).

Ad content featured images of young adults and mentioned the $15 gift card that eligible participants would receive if they completed the survey (Supplement Figure S1). Participants who clicked on these ads were directed to a link to a screener survey hosted on Qualtrics to assess eligibility. To be eligible, participants must be current smokers between 18 and 34 years of age. We also used conversion tracking whereby a pixel would inform Facebook that a person completed the screener and was eligible so that Facebook could use this information to target the ads to others more likely to be eligible and willing to participate.

Quotas were established to obtain a cigarette smoker sample of which approximately half were current LCC smokers and to ensure that gender and race/ethnicity distributions closely resembled national estimates for current smokers who use LCCs. After completing the screener, eligible participants provided informed consent and were taken to the study survey to complete the DCE and answer a series of tobacco use questions. Each participant who completed the survey received a $15 Amazon digital gift card, which was sent to participants via e-mail. Various procedures to prevent and detect fraudulent responses (e.g., requiring authentication with a Facebook account and checking for non-U.S.-based IP addresses and duplicate e-mail addresses) were implemented to ensure quality data. We also flagged extremely short response times, clusters of responses at or within certain times, and patterns of clustered responses. We further restricted the survey open time between 8 a.m. and 11:30 p.m. ET.

#### 2.1.2. Attributes

We chose cigarillo packaging-related attributes that are of regulatory interest, including product flavor (4 levels: regular, menthol, grape, wine), flavor depiction (3 levels: text only, color only, and color and text), text quality descriptors (“smooth,” “satisfying,” and “sweet”), pack size (4 levels: 1–4 sticks) and price per stick (3 levels: $0.5, $1, and $1.5). The levels of these attributes are popular ones observed in prior research and pilot research [15,22]. Table 1 summarizes the attributes and corresponding levels, as well as previous studies that inform the choices of attributes and their levels. The price levels reflect price per stick levels, which were rescaled based on pack sizes. For example, if price per stick was $0.5 and pack size was 2, the price of the product shown to participants was $1. Although this study was primarily motivated by generating evidence to inform FDA regulatory packaging policies that do not cover prices, we include prices as an attribute for the following two reasons: (1) prices can be used to convert non-price modeling parameters to the willingness to pay (e.g., the willingness to pay for a colored package), a comparable measure that can be used to gauge the importance of FDA policies regulating other packaging features; and (2) prices are an important determinant of LCC use behaviors among young adults who are price-sensitive, and will further inform state and local LCC pricing policies such as taxation.

#### 2.1.3. Choice Set Development

**Figure 1:**
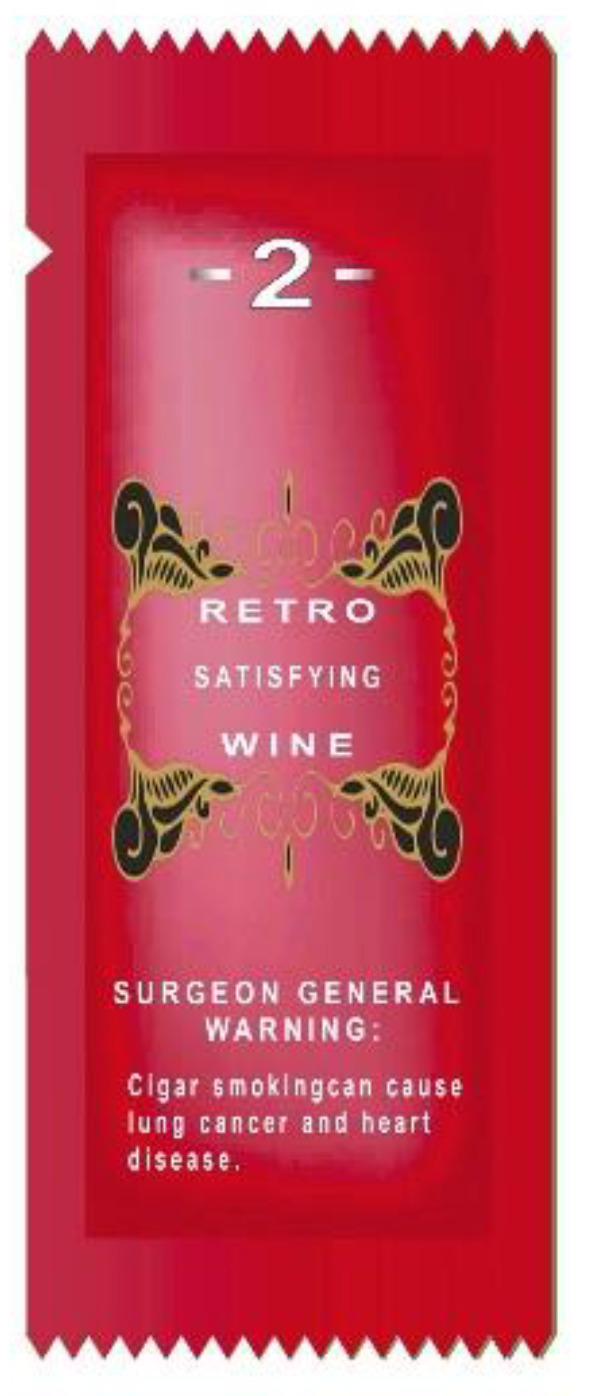
Hypothetical cigarillo package.

**Figure 2:**
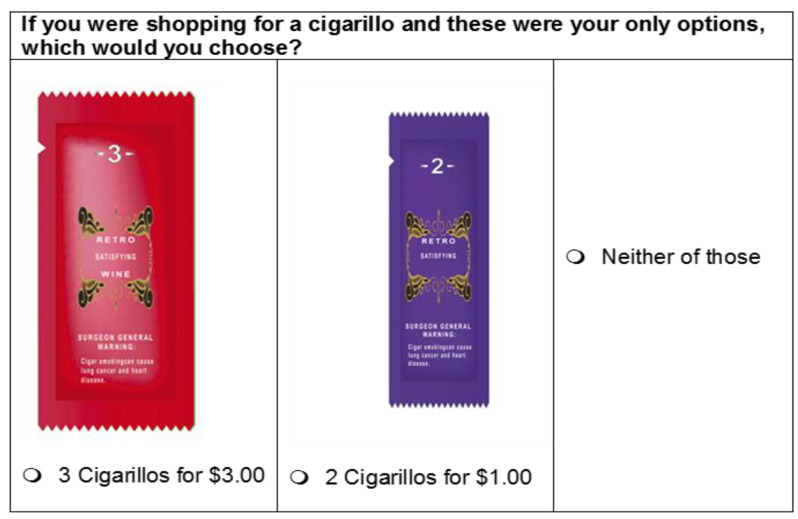
Sample choice set with hypothetical flavored cigarillo packages.

The number of attributes and their levels (4 flavors × 3 flavor depictions × 3 text descriptors × 4 pack sizes × 3 price levels) lead to 432 possible profiles. However, in the literature and practice, it is not recommended or feasible to present all choice combinations to respondents. [47] We used SAS JMP 13′s (SAS Institute Inc., Cary, NC, USA) choice experiment design module to select 60 choice sets of paired cigarillo options and one opt-out option based on a D-optimal design—an approach commonly used in choice modeling to select a subset of all possible choice combinations that optimizes model identifications (i.e., minimizing the generalized variance of the parameter estimates). This step is necessary to eliminate choice combinations that do not bring in new information (e.g., sets containing a dominant option) and select preferable ones that reaches a high modeling efficiency. [47] These 60 choice sets were further divided into 5 blocks, each containing 12 choice sets, to mitigate participants’ fatigue. Participants were randomly assigned to one of the 5 blocks. Figure 1 (A Hypothetical cigarillo package) shows a lower-resolution image of a cigarillo package and Figure 2 (Sample choice set with hypothetical flavored cigarillo packages) depicts an example choice set, which in the online survey were higher-resolution images of cigarillo packages than the image resolutions we present here. The online images were between 140 px and 185 px, depending on the package size; with minimal (~5 px or less) white space in the background. As shown in these figures, these package images did not represent any specific brand and all displayed a Surgeon General’s tobacco warning that was required by law at the time of the experiment.

### 2.2. Analyses

We used mixed (random parameter) logit regressions to jointly analyze the effects of package attributes on the choice of cigarillo, after controlling for individual-level sociodemographic characteristics (sex, age, race/ethnicity, income) and current combustible tobacco use status (current LCC use and menthol cigarette smoking). Standard errors were clustered at the individual level, since the analytical data contained repeated choices made by the same individual.

Random parameter logit (RPL) or mixed logit model analyses allow for a flexible assumption on the substitution pattern between the two alternative LCC products, thereby robust to the violation of independence of irrelevant alternative (IIA) assumption (i.e., preference for A over B does not depend on C), which may bias the estimation of product preference. This is an analytical advantage over other DCE analytical models such as nested or conditional logit that depend on the IIA assumption. It also allows for individual heterogeneity and thus will produce consistent estimates even when unobserved individual characteristics influence choice behaviors. The following equation was used to estimate the RPL model:

*Uij* = *α*1*i* PackSize*ij* + *α*2*i*
*Flavorij* + *α*3*i* FlavorDepic*ij* + *α*4*i* QualityDescript*ij* + *α*5*i*
*Priceij* + *βxi* + *εij*

where the four attribute variables are alternative specific (*ij*) while sociodemographic and tobacco use variables (*xi*) are individual specific (*i*).

In this equation, prices per stick (three levels: $0.5, $1 and $1.5) and pack size (1–4) are continuous variables, whereas characterizing flavors (grape as reference, menthol, tobacco/regular, and wine), flavor descriptions (text only as reference, color only, and text and color), and quality descriptors (“smooth” as reference, “satisfying,” and “sweet”) are reference-coded. The effect estimates of the attributes and their standard deviations, with the latter showing the heterogeneity in the attribute effects. All regressions further control for sociodemographic variables: gender (female vs. other), income (high: $50,000 or more, middle: $25,000–$49,999, and low: <$25,000 as reference), race (White, Black, and other/multi/missing race as reference group) and ethnicity (Hispanic vs. non-Hispanic) and combustible tobacco use status: menthol cigarette smoking in the past 30 days (Yes vs. no or missing) and current LCC use (smoke daily, smoke someday, and not at all). Specifically, current LCC use was measured using the question: “Do you now smoke cigarillos or filtered cigars every day, some days, or not at all?” Menthol cigarette smoking in the past 30 days was identified using the question: “Were most of the cigarettes you smoked in the last 30 days menthol?” Although there was a small proportion (2.8%) of respondents who did not report menthol smoking status, dropping them or grouping them with smokers who do not mostly smoke menthol cigarettes does not change results.

All regressions and simulations were conducted using Nlogit6 (Econometric Software, INC, Plainview, NY, USA) and a Broyden–Fletcher–Goldfarb–Shanno (BFGS) optimization algorithm.

Several sensitivity analyses were conducted. First, we analyzed data using nested and conditional logit regressions, which apply different assumptions about error structures. Second, we tested whether the inclusion or exclusion of combustible tobacco use status as control variables changes results. Third, we tested whether currently using an LCC product (a comparison of LCC users and non-users) moderates the effect of the different attributes on LCC choices, by including interactions between attributes and whether the participant is currently smoking LCCs. Fourth, we are analyzed the models using latent class analyses that assume heterogeneity in responses to attributes to be group-wise. Latent class analyses identified class memberships that may be distinguishable in their preference for tobacco products (e.g., number of classes, their shares, and characteristics that predict membership), as shown in adult cigarette smokers’ preference for e-cigarettes [36]. Last, we used pack prices instead of stick prices in the analyses.

## 3. Results

Supplement Table S1 shows the sociodemographic characteristics and tobacco use status and history of our study sample (*n* = 566). Participants were on average 28 years of age, and the majority were female, non-Hispanic, and White. The percentage of participants who had a household income <$25,000, $25,000–$49,999, and $50,000 or more was 35%, 30%, and 35%, respectively. Among our sample of young adult current cigarette smokers, 91% had used at least one of the following nicotine/tobacco products in their lifetime, even one or two puffs: hookah, e-cigarettes, and traditional cigars. Notably, 75% of our sample had ever smoked LCCs, even one or two puffs; and over half reported currently using LCCs (17% smoked LCCs daily and 35% smoked LCC some days). Menthol cigarettes smoking, measured as smoked mostly menthol cigarettes in the past 30 days, was 56%.

Table 2 shows results of analyzing choices using the RPL regression. In summary, young adults preferred grape over menthol, tobacco/regular, and wine flavors; “color only” and “color and text” flavor depictions over a text-only depiction; “smooth” and “sweet” LCC quality descriptors over “satisfying”; and bigger pack sizes and lower prices. Further, the RPL suggests that there is heterogeneity in respondents’ preference for menthol flavor (β = −0.25, β variance = 0.76, variance SE = 0.35) and for pack sizes (β = 0.06, β variance = 0.27, variance SE = 0.09).

Alternative specific constant estimates indicate that participants are likely to choose LCC products over opt-out, which is not surprising given that 75% of them had ever used LCCs before and over one-half currently used them. However, participants also preferred the second LCC product over the first one. This implies that participants tended to choose the product on the right over the one on the left when two products are shown horizontally side by side; and choose the bottom one over the top one when they are shown stacked vertically. Because the position or order of products did not differ systematically by design, the difference may be due to how choice sets were displayed.

Supplement Table S2 includes the full results of estimating conditional, nested, and random parameter or mixed logit models. The results are very similar across different modeling options. In addition, AICs and log-likelihood of the conditional, nested, and random parameter logit models are very close, suggesting that all three models fit the data well. We also find the following associations between sociodemographic characteristics and LCC preference: Among young adult smokers, the older they are, the less likely that they choose LCCs. Compared to other genders, female smokers are less likely to choose LCCs. Compared to Other/White/Multi races, Black smokers are less likely to choose LCCs. Compared with smokers with low or high income, those who fall into the middle-income category are more likely to choose LCCs. With regard to the associations between combustible tobacco use status and LCC preference, we find that those who reported smoking mostly menthol cigarettes in the past 30 days are less likely to choose LCCs; and those who are reported smoking LCCs some days or daily now are more likely to choose LCCs.

Supplement Table S3 includes data on the marginal WTPs of the packaging-related attributes using conditional logit results. The marginal WTP for greater pack sizes (WTP for having one additional stick in the pack) was $0.04 per stick. The marginal WTPs were $0.15–0.20 to choose packages that use color only or both color and text to depict flavors compared to packages that only use text. The marginal WTPs to choose a grape flavor over menthol, tobacco/regular, and wine flavors were $0.15, $0.17, and $0.18, respectively. The marginal WTP to choose packages with a “sweet” quality descriptor over those with a “smooth” descriptor was $0.06, and was $0.07 to choose those with a “smooth” compared to a “satisfying” descriptor. Based on these marginal WTP estimates, flavors and flavor depictions on packaging have the most pronounced effects on cigarillo preference among the packaging-related features that we studied.

Table 3 presents the changes in “market shares” (i.e., percentage of smokers who would choose LCCs over opting-out) under different simulated LCC packaging-related policy conditions. In these simulated scenarios, the sum of changes is set to be 0 (i.e., changes in market shares given the market size). In other words, the increase in market shares of LCCs is equivalent to the decrease in market shares of opting-out, and vice versa. Based on our benchmark model results, these simulations suggest that if the FDA banned grape and wine flavors, the share of young adult cigarette smokers who also smoke LCCs would drop by 0.87%. If the FDA banned grape, wine, and menthol flavors, the share of young adult cigarette smokers who also smoke LCCs would drop by 0.97%. If the FDA banned packages that depict flavors using colors (color only or color with text), the share of young adult cigarette smokers who also smoke LCCs would drop by 1.68%. If the FDA banned packages that have quality descriptors “sweet” and “smooth,” the share of young adult cigarette smokers who also smoke LCCs would drop by 0.85%.

Finally, we conducted a sensitivity analysis using both the latent class model and the stratified regressions by whether cigarette smokers are currently smoking LCCs. The findings are available by request and generally suggest no systematic differences in LCC preferences among our study sample. The latent class results identified two groups: one accounted for 98.1% of the sample and presents almost identical preferences for LCC attributes as described in the main findings; and the other accounted for only 1.9% of the sample, and is not responsive to any LCC attributes we present in this study. The AIC for the latent class model with two classes was smaller but nonetheless close to the AIC for the benchmark MNL model, further suggesting the validity of models without distinguishing latent classes. Models with additional interaction terms of current LCC use and attributes do not find any significant results for these interaction terms, suggesting that the preferences for LCCs do not differ by whether a cigarette smoker is using LCCs or not.

## 4. Discussion

To our knowledge, this is the first discrete choice experiment to examine the effects of packaging-related features on LCC preference among young adults in the US. Results show that young adult current cigarette smokers respond to several LCC packaging-related features and the product attributes they represent. In summary, young adult cigarette smokers prefer grape over menthol, tobacco/regular, and wine flavors in LCCs; prefer “color only” and “color and text” flavor depictions over a text-only flavor depiction; prefer “smooth” and “sweet” quality descriptors over “satisfying”; and prefer bigger pack sizes and lower prices. Further, there is no systematic difference in preference for these LCC attributes by current LCC smoking status (smokers vs. non-smokers).

Our study suggests that regulating flavor-related features may have the most pronounced impact on LCC smoking among potential packaging-related regulations. In the simulated scenarios, if the FDA banned packages that depict flavors using colors (color only or color with text), the share of young adult cigarette smokers who also smoke LCCs would drop by 1.68%. If the FDA banned grape, wine, and menthol flavors together, the share of young adult cigarette smokers who also smoke LCCs would drop by 0.97%. We also provide novel evidence that banning quality descriptors such as “sweet” and “smooth” could be effective in reducing LCC use; the share of young adult cigarette smokers who use LCCs would drop by 0.85% as a result of this ban.

Although our experiment did not explicitly specify the “opt-out” option, smokers may interpret this as choosing their own cigarettes or quitting. As a result, smokers may respond to LCC packaging regulations by either smoking more cigarettes or quitting. Future studies need to distinguish the nuances of these behaviors. Nonetheless, with more stringent cigarette regulations (e.g., increasing taxes) and increasing cessation services, policymakers have the opportunity to encourage quitting among young adults who are smoking or interested in smoking both cigarettes and LCCs.

We discovered important heterogeneities in young adult cigarette smokers’ preferences for LCC pack sizes and flavors. Specifically, our findings suggest that, although on average young adult cigarette smokers preferred grape over menthol flavors (β = −0.25), some smokers did prefer menthol over other flavors in LCCs (β variance = 0.76). This finding is consistent with other studies that also find heterogeneity in flavor preferences among cigarette smokers who are considering using e-cigarettes [36,40,42]. Although adult smokers on average prefer tobacco-flavored e-cigarettes, adult menthol cigarette smokers prefer menthol-flavored e-cigarettes [42]. Our study sample is not large enough to examine whether there is a similar reason for some young adult cigarette smokers to prefer menthol flavored LCCs, however nonetheless, we controlled for menthol cigarette smoking and still found the heterogeneity in LCC menthol flavor preference among this population. Moreover, the overall results suggest that grape flavors and thus likely fruit flavors and their color depictions have the most pronounced effects among packaging-related features on LCC preference. Therefore, regulating flavor-related features may be the most effective packaging-related regulation.

In addition, there is heterogeneity in young adult cigarette smokers’ preference for LCC pack size. While on average they prefer LCCs with bigger pack sizes (β = 0.06), some preferred smaller pack sizes (β variance = 0.27). The WHO Framework Convention on Tobacco Control advocates for minimum pack size regulation to make cigarette pack sizes larger (e.g., at least 20 sticks per pack) and less affordable to cigarette smokers. This is a sensible approach to make the price impact on smoking reduction more salient by increasing costs or expenditures of smoking. However, when price impact and pack size impact are considered separately, some cigarette smokers may prefer smaller cigarette pack sizes as a self-control mechanism to quit smoking [45]. Similarly in our study, we distinguished the effect of LCC pack size from the effect of prices on LCC preference. From a cost-minimizing perspective, smokers prefer smaller pack sizes and thus more affordable LCCs because they are price sensitive. However, once price impacts are accounted for, on average smokers prefer larger pack sizes. Our study also adds the evidence that, once price effects are accounted for, a portion of young adult cigarette smokers preferred smaller pack sizes. It is possible that these smokers use the smaller LCC pack size as a self-control mechanism to quit smoking. According to the 2013–2014 PATH survey, among adult cigarette smokers who are currently using LCCs, 15.6%–19.1% cited “smoking LCCs helps people to quit smoking cigarettes overall” and 28.2%–29.8% cited “I smoke them as a way to cut down on cigarette smoking” as a reason for smoking LCCs [6].

Young adult cigarette smokers are also very responsive to prices. Our study estimates the price elasticity of LCC demand to be −1.55. That is, a 10% increase in prices would reduce the demand for LCCs among young adult smokers by 15.5%. This finding is very similar to the existing evidence on the price elasticity of demand for little cigars and cigarillos, estimated using Nielsen Retailer Scanner data [46]. That study suggests the price elasticity of demand to be −1.665 for little cigars and −1.331 for cigarillos. The combined evidence suggests that the demand for LCCs is very sensitive and elastic to price increases and that increasing LCC taxes and prices would result in decreases in tax revenues collected from LCCs albeit reducing demand. Moreover, the price elasticity estimate from our hypothetical discrete choice experiment falls in the narrow elasticity range estimated using observational data, which is a validation of our study design and our estimates of the effects of LCC packing features on LCC preference [18].

Discrete choice experiments are increasingly used in tobacco control research to illuminate individual preferences for various tobacco products based on policy-relevant characteristics [36,37]. Gathering this preference data prior to regulatory action can help inform policy prior to its implementation. As such, findings from this study have implications for regulatory policy on LCCs. First, the experiment offers support for the FDA’s stated intention to ban characterizing flavors in LCCs [24], consistent with previous research findings that flavors increase product appeal and contribute to perceptions of lowered risk [8,14,22]. This result is particularly interesting within the current regulatory environment, as we await the emergence of research documenting the effects of the policy banning flavors in e-cigarettes. Second, this study provides evidence that packaging-related characteristics, including flavor and quality descriptors and pack size, could be manipulated to make LCCs less appealing to young adults. Finally, as the results indicated that the participants were more likely to choose products with lower price tags, policies designed to raise prices on LCCs also may have a public health impact.

This study is not without limitations. The DCE method carries an inherent risk of hypothetical bias. However, the price elasticity estimate for LCC demand in our study is very close to that estimated using observational data, which provides confidence in the results for other packaging-related features. The participants were a convenience sample recruited through a social media platform, and thus not demographically representative of all US young adults; however, the aim was to obtain choice preferences among young adult smokers, and further justified by the evidence that many LCC smokers are also smoking cigarettes. Additionally, the participants were cigarette smokers who had a history of or were current LCC users. Our findings may not generalize to other young adult smoking samples (e.g., LCC-only smokers). Future studies that use representative samples will address this limitation and better predict the market share shifts of different tobacco products in response to LCC regulation. In addition, this study only manipulated the attributes of LCCs and as a result we cannot explicitly estimate how cigarette regulation will impact LCC use and preference (e.g., whether a menthol cigarette ban will drive menthol cigarette smokers to smoking LCCs). Future studies are needed to understand cross-product policy impacts.

## 5. Conclusions

Regulating product attributes, including flavors, packaging-related features related to flavors (e.g., color packaging) and quality, pack size, and prices will have an impact on LCC choices among young adult cigarette smokers in the US. Specifically, eliminating grape flavor, color depictions of flavors, and “smooth” and “sweet” quality descriptors from LCC packages, increasing prices, and reducing pack sizes may on average reduce the probability of choosing LCCs among young adult cigarette smokers. Further, regulating flavor and pack sizes of LCCs requires additional caution, as they may impact young adult cigarette smokers who have different preferences for these attributes differently. Those who prefer smaller pack size will be more likely to choose LCCs if pack size is reduced, and those who prefer menthol to grape flavors will be more likely to choose LCCs if the grape flavor is removed. Finally, in addition to LCC regulations, more stringent cigarette regulations (e.g., increasing taxes) and increasing cessation services are needed to encourage quitting among young adults who are smoking or interested in smoking both cigarettes and LCCs.

## Figures and Tables

**Table 1:** Product attributes and levels.

Product Attribute	Attribute Levels	Selected Attribute Justification
Flavors	Tobacco/regular Menthol Grape Wine	Chen-Sankey et al. [43]; Mead et al. [44]; Meernik et al. [18], Nyman et al. [9]; Sterling et al. [15]
Flavor depiction	Text only Color only Color and text	Chen-Sankey et al. [43]; Meernik et al. [18], Mead et al. [44]; Nyman et al. [9]; Sterling et al. [15]
Text quality descriptors	Satisfying Smooth Sweet	Mead et al. [44]; Nyman et al. [9]
Pack size	Single 2 per pack 3 per pack 4 per pack	Mead et al. [44]; Meernik et al. [18], Corey et al. [6], Marti [45]
Price per stick	$0.50, $1.00, $1.50	Huang et al. [46]; Mead et al. [44]; Nyman et al. [9]

**Table 2:** The impacts of cigarillo package attributes on cigarillo choices using random parameter logit regressions.

	β (SE)	SD (SE)
LCC 2 (base)		
ASC: LCC 1	−0.084 ** (0.03)	--
ASC: Opt-out	−4.01 *** (0.4)	--
Descriptor		
Smooth (base)		
Satisfying	−0.1 *** (0.04)	0.07 (0.03)
Sweet	0.1 ** (0.05)	0.43 (0.6)
Flavor descriptor		
Text only (base)		
Color only	0.23 *** (0.04)	0.01 (0.08)
Color and Text	0.3 *** (0.05)	0.1 (0.12)
Flavor		
Grape (base)		
Menthol	−0.25 *** (0.06)	0.76 ** (0.35)
Tobacco/Regular	−0.28 *** (0.05)	0.01 (0.05)
Wine	−0.27 *** (0.06)	0.16 (0.21)
Other attributes		
Pack size	0.06 * (0.02)	0.27 *** (0.09)
Price	−1.55 *** (0.10)	0.05 (0.07)
Log likelihood	−5785	
AIC		

Note: *** *p* < 0.01, ** *p* < 0.05., * *p* < 0.1. Standard errors (SE) were clustered at the individual level. Regressions also control for sex, age, race/ethnicity, income, current use of LCCs (daily, someday, or not at all), and past-30-day menthol smoking (Yes vs. no/missing). SEs were clustered by ID.

**Table 3:** Policy impact simulation of potential LCC packaging regulation.

Scenario 1: Banning Grape and Wine Flavors (e.g., Flavors Other than Menthol or Tobacco)			
	Base	Scenario	Change
LCC	90.45%	89.58%	−0.87%
Opt-out	9.55%	10.42%	0.87%
Scenario 2: Banning grape, wine, and menthol flavors (e.g., flavors other than tobacco)			
	Base	Scenario	Change
LCC	90.45%	89.48%	−0.97%
Opt-out	9.55%	10.52%	0.97%
Scenario 3: Banning color flavor depictions (color only or color with text)			
	Base	Scenario	Change
LCC	90.45%	88.77%	−1.68%
Opt-out	9.55%	11.23%	1.68%
Scenario 4: Banning quality descriptors “sweet” and “smooth”			
	Base	Scenario	Change
LCC	90.45%	41.08%	−0.85%
Opt-out	9.55%	48.51%	0.85%

## Data Availability

Data are available by request.

## Supplementary Materials

The following are available online at https://www.mdpi.com/article/10.3390/ijerph182111443/s1. Table S1. Summary Statistics (N = 566). Table S2. Results (N = 6765). Table S3. Marginal willingness to Pay (WTP) based on MNL model estimates (N = 6765). Figure S1. Facebook recruitment advertisement.

## References

[1] Alcohol and Tobacco Tax and Trade Bureau (TTB) (2019). Tobacco Statistics.

[2] Rostron B.L., Cheng Y.-C., Gardner L.D., Ambrose B.K. (2020). Prevalence and reasons for use of flavored cigars and ENDS among US youth and adults: Estimates from Wave 4 of the PATH Study, 2016–2017. Am. J. Health Behav..

[3] Campaign for Tobacco-Free Kids (2019). The Rise of Cigars and Cigar-Smoking Harms.

[4] National Cancer Institute (1998). Cigars: Health Effects and Trends. Smoking and Tobacco Control Monograph No. 9. Bethesda.

[5] Chaloupka F.J., Straif K., Leon M.E. (2011). Effectiveness of tax and price policies in tobacco control. Tob. Control..

[6] Corey C.G., Holder-Hayes E., Nguyen A.B., Delnevo C.D., Rostron B.L., Bansal-Travers M., Kimmel H.L., Koblitz A., Lambert E., Pearson J.L. (2018). US adult cigar smoking patterns, purchasing behaviors, and reasons for use according to cigar type: Findings from the Population Assessment of Tobacco and Health (PATH) Study, 2013–2014. Nicotine Tob. Res..

[7] Klein S.M., Giovino G.A., Barker D.C., Tworek C., Cummings K.M., O’Connor R.J. (2008). Use of flavored cigarettes among older adolescents and adult smokers: United States, 2004–2005. Nicotine Tob. Res..

[8] Kostygina G., Glantz S.A., Ling P.M. (2016). Tobacco industry use of flavours to recruit new users of little cigars and cigarillos. Tob. Control.

[9] Nyman A.L., Sterling K.L., Weaver S.R., Majeed B.A., Eriksen M.P. (2016). Little cigars and cigarillos: Users, perceptions, and reasons for use. Tob. Regul. Sci..

[10] Antognoli E., Gonzalez S.K., Trapl E., Cavallo D., Lim R., Lavanty B., Flocke S. (2018). The social context of adolescent co-use of cigarillos and marijuana blunts. Subst. Use Misuse.

[11] Cohn A.M., Johnson A.L., Fryer C.S., Villanti A.C. (2018). Marijuana use predicts onset of current little cigar use in a national sample of US young adults. Drug Alcohol Depend..

[12] Gonzalez S.J.K., Cofie L.E., Trapl E.S. (2017). I just use it for weed: The modification of little cigars and cigarillos by young adult African American male users. J. Ethn. Subst. Abus..

[13] Regan A.K., Dube S.R., Arrazola R. (2012). Smokeless and flavored tobacco products in the U.S.: 2009 Styles Survey results. Am. J. Prev. Med..

[14] Nyman A.L., Sterling K.L., Majeed B.A., Jones D.M., Eriksen M.P. (2018). Flavors and risk: Perceptions of flavors in little cigars and cigarillos among U.S. adults, 2015. Nicotine Tob. Res..

[15] Sterling K.L., Fryer C.S., Fagan P. (2016). The most natural tobacco used: A qualitative investigation of young adult smokers’ risk perceptions of flavored little cigars and cigarillos. Nicotine Tob. Res..

[16] Kong G., Cavallo D., Bold K., LaVallee H., Krishnan-Sarin S. (2017). Adolescent and young adult perceptions on cigar packaging: A qualitative study. Tob. Regul. Sci..

[17] Lempert L.K., Glantz S. (2017). Packaging colour research by tobacco companies: The pack as a product characteristic. Tob. Control..

[18] Meernik C., Ranney L.M., Lazard A.J., Kim K., Queen T.L., Avishai A., Boynton M.H., Sheeran P.J., Goldstein A.O. (2018). The effect of cigarillo packaging elements on young adult perceptions of product flavor, taste, smell, and appeal. PLoS ONE.

[19] Nonnemaker J.M., Pepper J.K., Sterling K.L., Kemp C.B., Taylor N., Bradfield B.R., Kim A.E. (2018). Adults’ visual attention to little cigar and cigarillo package warning labels and effect on recall and risk perceptions. Tob. Regul. Sci..

[20] Cohn A., Cobb C.O., Niaura R.S., Richardson A. (2015). The other combustible products: Prevalence and correlates of little cigar/cigarillo use among cigarette smokers. Nicotine Tob. Res..

[21] Messer K., White M.M., Strong D.R., Wang B., Shi Y., Conway K.P., Pierce J.S. (2015). Trends in use of little cigars or cigarillos and cigarettes among U.S. smokers, 2002–2011. Nicotine Tob. Res..

[22] Sterling K.L., Fryer C.S., Nix M., Fagan P. (2015). Appeal and impact of characterizing flavors on young adult small cigar use. Tob. Regul. Sci..

[23] Corey C.G., King B.A., Coleman B.N., Delnevo C.D., Husten C.G., Ambrose B.K., Apelberg B.J. (2014). Little filtered cigar, cigarillo, and premium cigar smoking among adults—United States, 2012–2013. MMWR Morb. Mortal. Wkly. Rep..

[24] FDA (2018). Press Announcement—Statement from FDA Commissioner Scott Gottlieb, M.D., on Proposed New Steps to Protect Youth by Preventing Access to Flavored Tobacco Products and Banning Menthol in Cigarettes. http://www.fda.gov/news-events/press-announcements/statement-fda-commissioner-scott-gottlieb-md-proposed-new-steps-protect-youth-preventing-access.

[25] Countertobacco.org (2018). FDA Announces Proposed Ban on Menthol Cigarettes and Flavored Cigars, Restrictions on Flavored e-Cigarettes. https://countertobacco.org/fda-announces-proposed-ban-on-menthol-cigarettes-and-flavored-cigars-restrictions-on-flavored-e-cigarettes/.

[26] Food and Drug Administration C for TP (2019). Questions on FDA’s Deeming Regulations for e-Cigarettes, Cigars, and all Other Tobacco Products.

[27] Food and Drug Administration (2020). Enforcement Priorities for Electronic Nicotine Delivery Systems and Other Deemed Products on the Market Without Premarket Authorization; Guidance for Industry; Availability. Fed. Regist..

[28] Pinilla J., López-Valcárcel B.G., Negrín M.A. (2019). Impact of the Spanish smoke-free laws on cigarette sales, 2000–2015: Partial bans on smoking in public places failed and only a total tobacco ban worked. Health Econ. Policy Law.

[29] Moodie C., MacKintosh A.M., Brown A., Hastings G.B. (2018). Tobacco marketing awareness on youth smoking susceptibility and perceived prevalence before and after an advertising ban. Eur. J. Public Health.

[30] Almeida A., Galiano A., Golpe A.A., Martín-Álvarez J.M. (2021). The Usefulness of Marketing Strategies in a Regulated Market: Evidence from the Spanish Tobacco Market. EM Econ. Manag..

[31] Buckell J., Hess S. (2019). Stubbing out hypothetical bias: Improving tobacco market predictions by combining stated and revealed preference data. J. Health Econ..

[32] Nasim A., Guy M.C., Soule E.K., Cobb C.O., Blank M.D., Eissenberg T. (2016). Characteristics and patterns of Black & Mild use among African American smokers. Nicotine Tob. Res..

[33] Trapl E.S., Yoder L.D., Frank J.L., Borawski E., Sattar A. (2016). Individual, parental, and environmental correlates of cigar, cigarillo, and little cigar use among middle school adolescents. Nicotine Tob. Res..

[34] Sterling K.L., Fryer C.S., Pagano I., Fagan P. (2016). Little cigars and cigarillos use among young adult cigarette smokers in the United States: Understanding risk of concomitant use subtypes. Nicotine Tob. Res..

[35] Shang C., Huang J., Chaloupka F.J., Emery S.L. (2018). The impact of flavour, device type and warning messages on youth preferences for electronic nicotine delivery systems: Evidence from an online discrete choice experiment. Tob. Control..

[36] Buckell J., Sindelar J.L. (2019). The impact of flavors, health risks, secondhand smoke and prices on young adults’ cigarette and e-cigarette choices: A discrete choice experiment. Addiction.

[37] de Bekker E.W., Ryan M., Gerard K. (2012). Discrete choice experiments in health economics: A review of the literature. Health Econ..

[38] Regmi K., Kaphle D., Timilsina S., Tuha N.A.A. (2018). Application of discrete-choice experiment methods in tobacco control: A systematic review. Pharm. Open.

[39] Louviere J.J., Hensher D.A., Swait J.D. (2000). Stated Choice Methods: Analysis and Application.

[40] Marti J., Buckell J., Maclean J.C., Sindelar J. (2019). To “vape” or smoke? Experimental evidence on adult smokers. Econ. Inq..

[41] Shi Y., Cao Y., Shang C., Pacula R.L. (2019). The impacts of potency, warning messages, and price on preferences for Cannabis flower products. Int. J. Drug Policy.

[42] Shang C., Weaver S.R., White J.S., Huang J., Nonnemaker J., Cheng K.-W., Chaloupka F.J. (2020). E-cigarette product preferences among adult smokers: A discrete choice experiment. Tob. Regul. Sci..

[43] Chen-Sankey J.C., Choi K., Kirchner T.R., Feldman R.H., Butler J., Mead E.L. (2019). Flavored cigar smoking among African American young adult dual users: An ecological monetary assessment. Drug Alcohol Depend..

[44] Mead E.L., Johnson S.L., Siddiqui J., Butler J., Kirchner T., Feldman R.H. (2018). Beyond blunts: Reasons for cigarette and cigar use among African American young adult dual users. Addict. Res. Theory.

[45] Marti J., Sindelar J. (2015). Smaller cigarette pack as a commitment to smoke less? Insights from behavioral economics. PLoS ONE.

[46] Huang J., Gwarnicki C., Xu X., Caraballo R.S., Wada R., Chaloupka F.J. (2018). A comprehensive examination of own- and cross-price elasticities of tobacco and nicotine replacement products in the U.S. Prev. Med..

[47] Hensher D.A., Rose J.M., Rose J.M., Greene W.H. (2005). Applied Choice Analysis: A Primer.

