# Supplementary material for: Impact of Little Cigars and Cigarillos Packaging Features on Product Preference
Source: Int J Environ Res Public Health. 2021 Oct 30;18(21):11443. doi: 10.3390/ijerph182111443 (PMC8583443; doi:10.3390/ijerph182111443)
Supplement: Supplementary file 1 [file ijerph-18-11443-s001.zip › ijerph-1394244-supplementary.pdf]

Table S1. Summary Statistics (N=566).

Variables	Mean/%	Std. Dev.
Demographics		
Age (18-34)	27.96	4.08
Female	63%	0.48
Race/Ethnicity		
Hispanic	19%	0.39
White	71%	0.45
Black	15%	0.36
Other/multi races	12%	0.32
Missing race	2%	0.14
Income		
Income: Low < $25,000	35%	0.48
Income: Middle $25,000-$49,999	30%	0.46
Income: High $50,000 or more	35%	0.48
Tobacco use		
Ever used LCC	75%	0.43
Current use of LCC	52%	0.50
Daily use of LCC	17%	0.38
Someday use of LCC	35%	0.48
Menthol cigarette smoking	56%	0.50

Table S2. Results (N=6,765).

	MNL β(se)	NL β(se)	RPL β(se)	RPL SD(se)
LCC 2 (base)				
ASC: LCC 1	-0.07** (0.03)	-0.03* (0.02)	-0.09** (0.03)	--
ASC: Opt out	-3.87 (2.46)	-3.61***(0.37)	-4.01***(0.4)	--
Descriptor				
Smooth (base)				
Satisfying	-0.1*** (0.03)	-0.06*** (0.02)	-0.1*** (0.04)	0.06(0.09)
Sweet	0.09* (0.05)	0.04 (0.03)	0.1** (0.05)	0.41 (0.58)
Flavor Descriptor				
Text only (base)				
Color only	0.21*** (0.05)	0.12*** (0.03)	0.23*** (0.04)	0.01 (0.08)
Color and Text	0.29*** (0.05)	0.16*** (0.04)	0.3*** (0.05)	0.1 (0.13)
Flavors				
Grape (base)				
Menthol	-0.21*** (0.08)	-0.12*** (0.03)	-0.25*** (0.06)	0.84*** (0.32)
Tobacco/Regular	-0.25*** (0.08)	-0.14*** (0.04)	-0.29*** (0.05)	0.003 (0.05)
Wine	-0.25***(0.07)	-0.15***(0.04)	-0.27***(0.06)	0.13(0.19)
Other pack attributes				
Pack size	0.05*(0.03)	0.03***(0.01)	0.06***(0.02)	0.27***(0.09)
Price	-1.42*** (0.13)	-0.79*** (0.15)	-1.56*** (0.09)	0.05 (0.07)
Demographics				
Age	-0.04 (0.08)	-0.04***(0.01)	-0.05***(0.01)	--
Female	-0.21 (0.67)	-0.22** (0.1)	-0.22**(0.1)	--
Race				
Other/multi/missing race (base)				
White	0.20 (0.89)	0.19 (0.14)	0.22(0.15)	--
Black	-0.34 (1)	-0.35** (0.17)	-0.35** (0.17)	--
Ethnicity				

Hispanic vs. nonHispanic	0.19 (0.76)	0.19 (0.12)	0.21(0.13)	--
Income				
Low (base)				
Middle	0.29 (0.63)	0.28** (0.11)	0.31***(0.12)	--
High	0.04 (0.71)	0.03 (0.1)	0.04(0.11)	--
Tobacco use				
Smoking menthol cigarettes vs. others	-0.23 (0.61)	-0.23**(0.09)	-0.27***(0.1)	--
Not smoking LCCs (base)				
Smoking LCCs some days	0.63 (0.61)	0.62***(0.1)	0.68***(0.11)	--
Smoking LCCs daily	0.71 (0.75)	0.69***(0.13)	0.75***(0.14)	--
IV (lcc)	--	0.53***(0.1)		
SD (lcc)	--	2.42***(0.47)		
Log Likelihood	-5784	-5775	-5781	
AIC	11611	11594	11624	

Note: *** $p < 0.01$, ** $p < 0.05$, *p<0.1. Standard errors (S.E) were clustered at the individual level. SEs were clustered by ID. MNL-Multinomial Logit regression; NL-Nested Logit regression; RPL- Random Parameter Logit regression.

Table S3. Marginal willingness to Pay (WTP) based on MNL model estimates (N=6,765).

Quality Descriptor.		
Smooth (base)		
Satisfying	-0.07***	0.02
Sweet	0.06*	0.03
Flavor Depiction		
Text only (base)		
Color only	0.15***	0.04
Color and Text	0.2***	0.04
Flavors		
Grape (base)		
Menthol	-0.15**	0.06
Tobacco/Regular	-0.17***	0.06
Wine	-0.18***	0.05
Pack size	0.04*	0.02

Note: *** $p < 0.01$, ** $p < 0.05$, *p<0.1.

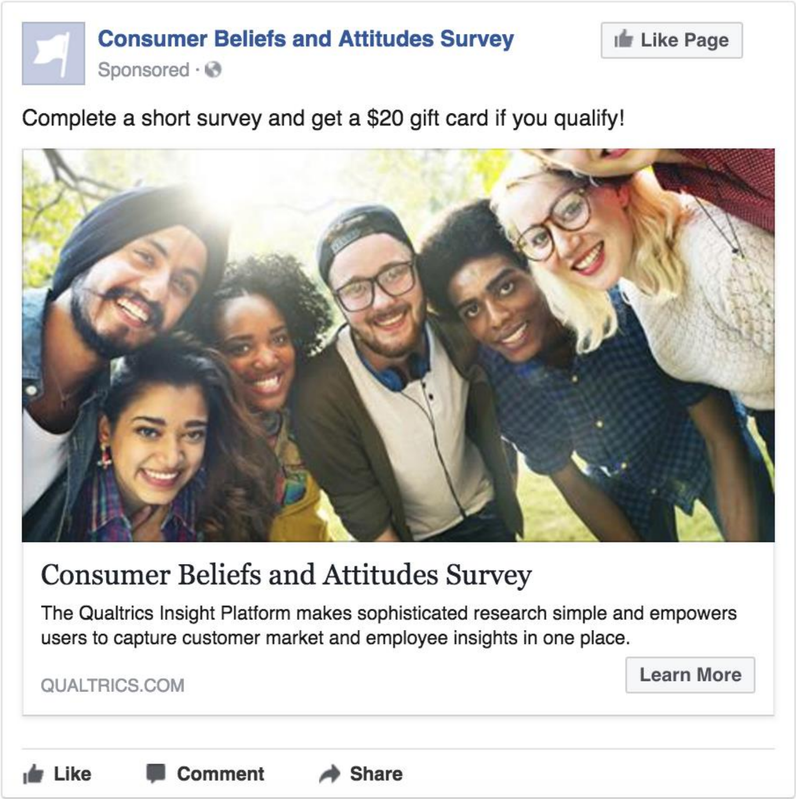

Figure S1 Facebook recruitment advertisement
